# Identifying neural mediators and cultural moderators of the association between discrimination and depression among Mexican American youth

**DOI:** 10.1038/s41598-025-22900-8

**Published:** 2025-11-06

**Authors:** Angelica F. Carranza, Sarah J. McMillan, Amanda E. Guyer, Paul D. Hastings, Richard W. Robins, Johnna R. Swartz

**Affiliations:** 1 Department of Human Ecology, University of California Davis, Davis, CA USA; 2 Department of Psychology, University of California Davis, Davis, CA USA; 3 Center for Mind and Brain, University of California Davis, Davis, CA USA

**Keywords:** Adolescence, fMRI, Depression, Discrimination, Ethnic pride, Familism, Neuroscience, Psychology

## Abstract

Prior research has demonstrated a link between perceived discrimination during adolescence and depression symptoms, but few studies have examined whether this link holds into young adulthood. Moreover, little is known about the neural mechanisms that explain the association between discrimination and depression, or the protective cultural factors that buffer youth against the adverse effects of discrimination. These gaps were addressed with a sample of 196 Mexican-origin youth (51% female) enrolled in the longitudinal California Families Project. Perceived discrimination was assessed in early adolescence (ages 10–14); participants completed two neuroimaging scans while experiencing social exclusion in late adolescence (ages 16 and 18); and, depression symptoms were assessed in young adulthood (ages 21 and 23). Growth curve analyses tested whether perceived discrimination was associated with depression symptoms in young adulthood, whether exclusion-related neural activity mediated this association, and whether cultural factors (ethnic pride, familism) moderated these associations. No significant effects were found, except that higher levels of ethnic pride in later adolescence were associated with lower levels of depression symptoms in young adulthood ($\beta$ = − .17, *SE* = .06, *p* = 0.007). Findings suggest that ethnic pride could be leveraged in interventions to promote more positive mental health outcomes in Mexican-origin individuals in young adulthood.

**Supplementary Information:**

The online version contains supplementary material available at 10.1038/s41598-025-22900-8.

## Introduction

Experiences of discrimination among racial/ethnic minorities can increase risk for developing depression^1,2^. Yet underlying explanatory mechanisms and factors that protect against depression in racial/ethnic minorities have received little research attention^3^, despite their ability to inform interventions for depression. One plausible explanatory mechanism involves how the brain processes discrimination experiences, particularly during adolescence when the brain is still developing and highly sensitive to social stressors^4^. Prior research suggests that racial discrimination (which involves experiences of exclusion) influences brain function during social exclusion in adults^5^. Research also shows that altered brain function associated with social exclusion increases risk for depression in adolescents^6,7^. However, relatively little research has examined perceived discrimination, exclusion-related brain function, and depression symptoms within one study and longitudinally over the course of adolescence. Furthermore, positive cultural factors (e.g., strong ethnic pride) and values (e.g., familism; strong family relationships, unity and cohesion within the family) may mitigate the adverse effects of discrimination on mental health^8,9^, but have not been examined as potential moderators of brain pathways of depression risk. The objective of the present study is to address these research gaps by examining how trajectories of perceived discrimination across adolescence are associated with social exclusion-related brain function in late adolescence and depression symptoms in young adulthood, as well as how cultural factors (ethnic pride and familism) may moderate these associations in Mexican–American individuals.

Latino youth are expected to comprise more than a quarter of the US population by 2060^10^ and at present, 60% of the Latino population in the US is of Mexican origin^11^. Given that Mexican–American youth face higher rates of perceived discrimination and associated health disparities relative to their Caucasian counterparts^12^, and that Latino youth are projected to become one of the largest racial/ethnic minority groups in the US, with Mexican-Americans comprising the largest share, this population of youth warrants particular attention. Doing so will be crucial to deepen our understanding of the effects of racial inequality on developmental outcomes to better inform efforts aimed at promoting positive mental health outcomes for these youth.

### Effects of discrimination on depression in Latino youth

Minoritized youth have been found to develop an increased awareness of racial inequality during adolescence^13^. Among Mexican-origin youth in the United States (US), by the age of 16, almost three-quarters (74%) report believing Latinos are viewed negatively by society^14^. Experiencing racial inequality (e.g., discrimination) also has effects on developing depression in adolescence. For Latino adolescents, experiences with discrimination in 9th grade (age 14–15) confer risk for developing depression in 11th grade (age 16–17)^1^ and discrimination experienced between 17 and 18 is positively associated with depression at this same age^15^. Experiences with discrimination also increase psychological distress experienced by 15–17 year-old Latinos^16^. Among Mexican-origin youth in particular, discrimination experienced at age 14 predicts more depression symptoms at age 17^2,17^. Given the documented effect of discrimination on depression in Latino adolescents, further work is necessary to uncover mechanisms underlying the ways in which discrimination conveys risk for developing depression in young adulthood and how important cultural values (e.g., familism) may weaken the effects of discrimination on neurobiology and depression.

One such mechanism is how the brain processes social exclusion, given consistent evidence for its connection with depression in adolescents and adults^7,18–20^. Racial discrimination operates through unconscious biases of superiority, enabling the dominant racial group to disempower those perceived as inferior through an unequal distribution of resources and opportunities^21^. Racial discrimination, therefore, is not only an isolated experience but functions as a form of social exclusion by denying individuals full participation in society. This exclusion operates at both structural and individual levels. At the structural level, racism functions within and across societal and cultural domains as well as through laws, policies, and practices that create social inequality and can exclude individuals from access to resources and opportunities^21^. At the individual level, racial discrimination functions within one’s immediate environment, operating through interpersonal prejudice and unfair treatment from members of the dominant racial group, resulting in exclusion from social groups and interactions that foster a sense of belonging^21^. Such experiences of discrimination are deeply personal and prior neuroimaging findings suggest that these experiences may influence how the brain processes social exclusion.

### Experiences of discrimination associated with neural activity during social exclusion

In functional magnetic resonance imaging (fMRI) research, neural response to social exclusion is often measured with the Cyberball task, in which participants are either included or excluded from a simulated ball-toss game by other people^22,23^. Neural activity to social exclusion delivered via the Cyberball task is consistently elicited in brain regions including the anterior cingulate cortex (ACC), anterior insula (AI), prefrontal cortex (PFC), and amygdala^22,24^. The subgenual ACC (sgACC) and AI are associated with evaluating subjective distress of being excluded, the dorsal ACC (dACC) is important for conflict monitoring and emotion regulation, and the ventrolateral PFC (vlPFC) and dorsolateral PFC (dlPFC) are associated with regulating top-down control of subjective distress from being excluded^22,25^. It is also believed the ACC plays a particularly important role in exacerbating the negative effects of social exclusion due to its role in social pain processing^26^ and within the dACC, a role in anxiety and substance use^27^.

Previous work evaluating the neural correlates of social exclusion in adolescence and adulthood, using the Cyberball task, has found that social exclusion and self-reported distress are positively associated with brain activity in the sgACC and AI as well as negatively associated with vlPFC brain activity^25,26,28,29^. A previous study of social exclusion conducted with the California Families Project (the same sample as the current paper) found that hostile school environments (operationalized as perceived discrimination and school violence) experienced across ages 14 to 16 predicted increased neural activity within the rostral sgACC when being excluded versus included at age 17^30^. A different neuroimaging study examining the effects of discrimination in Black adults (aged 19–28 years old) also found that greater distress during social exclusion in the Cyberball task (rated by an observer) was associated with increased neural activity in the dACC and insula, and reduced neural activity in the PFC^5^. There is also some overlap in the association between discrimination and brain structure. Specifically, when examining gray matter volume in Black women (aged 19–62 years), more racial discrimination exposure was associated with lower cortical thickness of the ACC and posterior cingulate cortex^31^. Together, these findings suggest that experiences of discrimination influence how affectively salient neural regions respond to social exclusion.

### Neural activity during social exclusion in association with depression

During adolescence, youth experience significant maturation in neural regions necessary for social information processing, which may heighten sensitivity to social evaluation and feedback^32^,^33^,^34^. Adolescents may be especially vulnerable to social evaluation and negative feedback in the form of exclusion or rejection^35^, as both have been shown to impact depression outcomes ^6,7,33^. Furthermore, youth who are depressed or diagnosed with major depressive disorder (MDD) relative to non-depressed youth, show greater neural activity in the AI, sgACC, amygdala, and striatum to social rejection or exclusion ^7,33^. Prior neuroimaging studies focusing on the association between exclusion-related neural activity and depression have primarily been cross-sectional^6,7,33^; thus, it remains unknown from this cross-sectional work whether higher brain activation to social exclusion predicts depression longitudinally. However, longitudinal research in adults found that increases in self-reported ostracism (e.g., being ignored or socially excluded) were associated with greater depressive symptoms three years later^36^ and research using self-report measures of perceived discrimination has shown longitudinal associations with depression symptoms in adolescents^1,2,17^, suggesting that exclusion-related neural activity may be a potential mechanism explaining this longitudinal association with depression over time. Given that cross-sectional work has linked hyperactivity in exclusion-related brain regions to depression, and longitudinal work has linked self-reported experiences of discrimination and ostracism to depression, these findings suggest that experiences of racial discrimination during adolescence may lay a neurobiological pathway of risk for developing or sustaining depression through effects on how the brain processes social exclusion.

### Cultural factors that protect against development of depression in Latino youth

Among Latino youth, cultural protective factors (e.g., ethnic pride and familism) may also mitigate some of the associations between discrimination and mental health problems during adolescence^37^ but it is unclear how this protective effect extends into young adulthood. For instance, ethnic pride and familism are consistently associated with more positive adjustment, lower delinquency, and reduced depression and anxiety in adolescents^9,16,38–41^. Cultural protective factors may therefore provide an important promotive benefit for Latino youth at risk for developing depression, especially in the face of experiencing racial discrimination during the developmental period of adolescence, which we highlight further in the following sections.

For minoritized youth, including Latino adolescents, holding a positive view towards one’s racial and ethnic identity has been shown to affect a multitude of outcomes related to mental health^42^. Greater ethnic pride and endorsement of Mexican–American cultural values, for example, are associated with fewer depression symptoms and lower internalizing problems^43–45^. Moreover, when Latino youth held a positive view about their ethnic-racial background, levels of depressive symptoms decreased^8^, above any influence from ethnic discrimination. The extent to which an individual considers their race as an integral part of their self-concept has also dampened the negative effects of discrimination on psychological well-being^46^. Thus, an adolescent’s beliefs surrounding their ethnic-racial identity (ERI) can provide a clearer understanding for how race and ethnicity are interconnected with their sense of self and pride. A stronger connection between ERI and self-concept may alleviate the negative psychological effects of discrimination, including heightened risk for depression, as demonstrated in prior research. For example, among Latino youth, higher perceived ethnic group discrimination is associated with lower levels of how good one feels about being in their racial/ethnic group, and lower levels on this dimension are associated with higher depression symptoms^47–49^. Prior findings suggest that aspects of ethnic pride and identity play an important role in mitigating the effects of discrimination on depression symptoms throughout adolescence.

Another salient cultural factor for Latino youth is familism (*familismo* in Spanish), which refers to the importance of maintaining strong family relationships and promoting unity and cohesion within the family^50^. Latino youth who hold high familism values tend to exhibit increases in psychological well-being^51^, have better coping strategies in the face of stress^52^, have fewer daily internalizing symptoms^45,53^, and lowered likelihood of developing depression^9,41^. Longitudinal studies have also found that higher familism values at age 15 are associated with fewer internalizing symptoms at age 17^54^ and, that family connectedness interacts with hippocampal volumes to predict depression symptoms^55^. However, contrary to these findings, some studies have found that familism values do not always protect Mexican-origin youth from stressors related to discrimination. For instance, in one study, high levels of familism and family cohesion strengthened the association between discrimination and depression symptoms^17^. Then in another study of Mexican–American youth using the same sample from our study, familism was not associated with emotion regulation and anhedonia, two relevant correlates of depression^56^. More research is therefore needed to understand the ways in which familism may serve as a protective mechanism in the face of perceived discrimination and depression in Mexican-origin individuals.

### Study aims

**Fig. 1:**
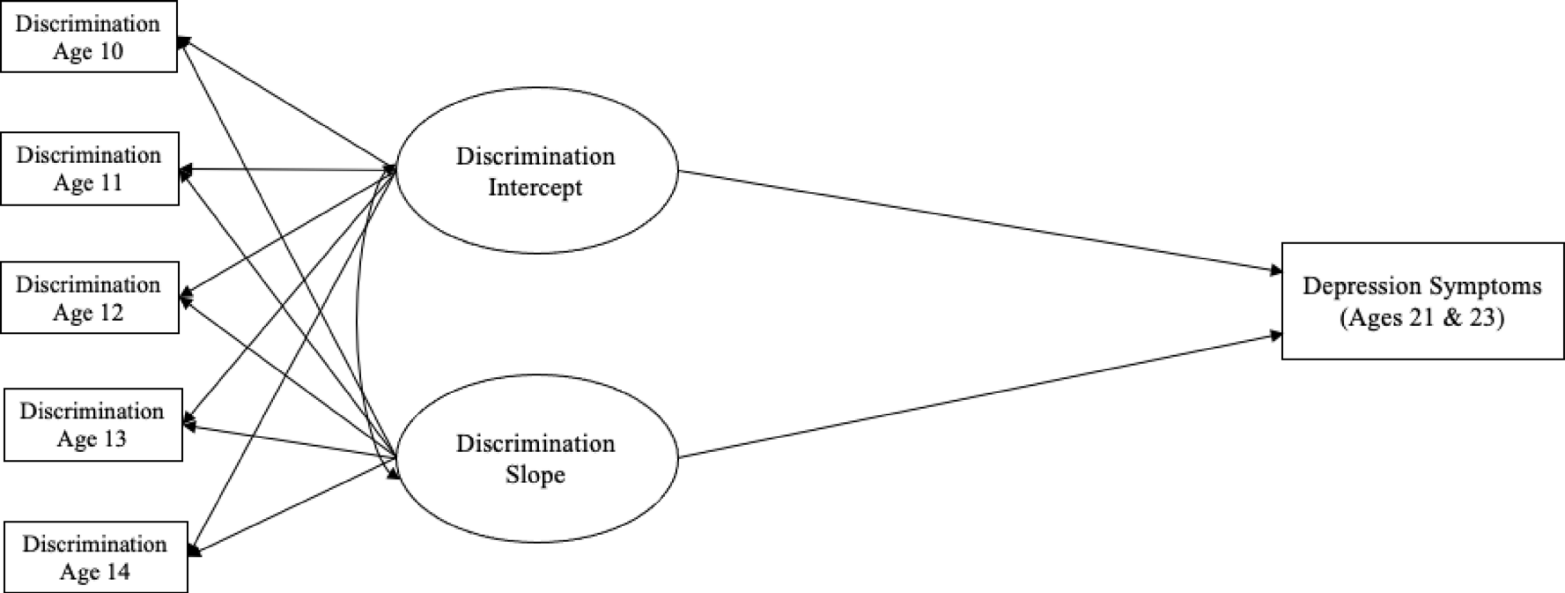
Growth curve model tested for associations between earlier perceived discrimination and depression symptoms in young adulthood. A latent basis growth curve model was used to test longitudinal associations between earlier perceived discrimination and later depression symptoms in young adulthood.

**Fig. 2:**
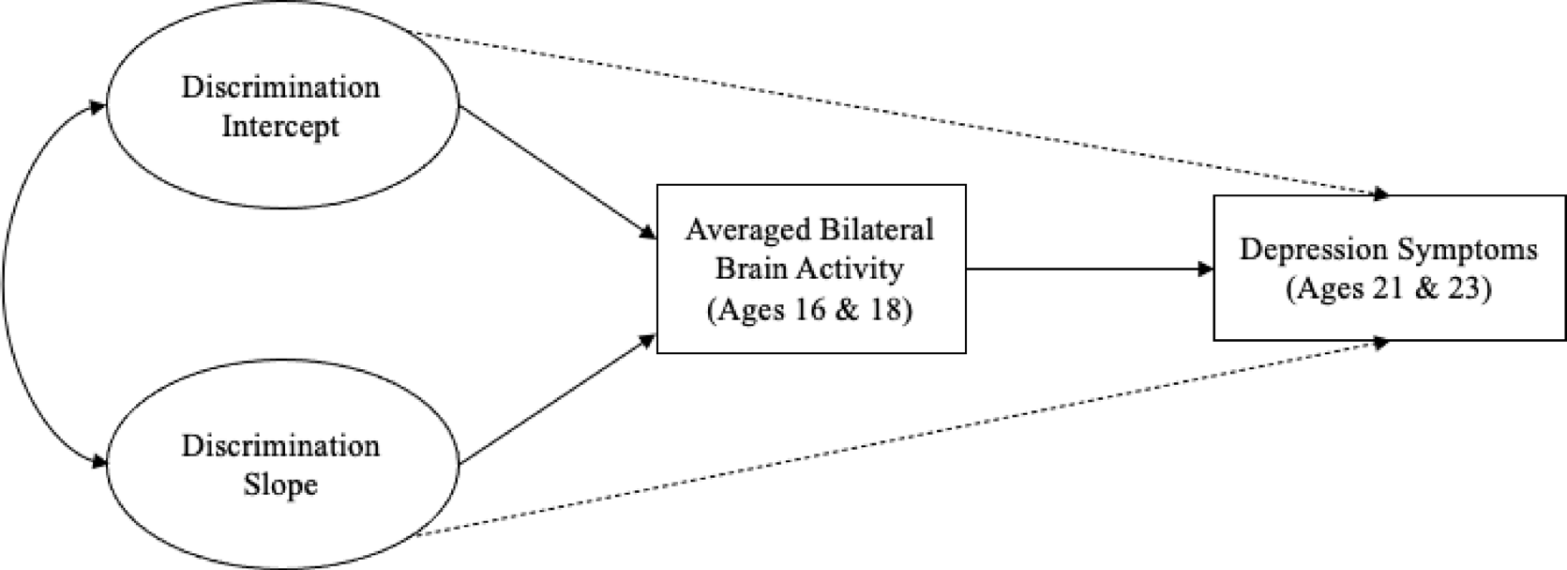
Growth curve model tested for mediating effect of brain activity. A latent basis growth curve model was used to test longitudinal associations between earlier perceived discrimination and later depression symptoms in young adulthood, with averaged brain activity in later adolescence as a mediating variable.

**Fig. 3:**
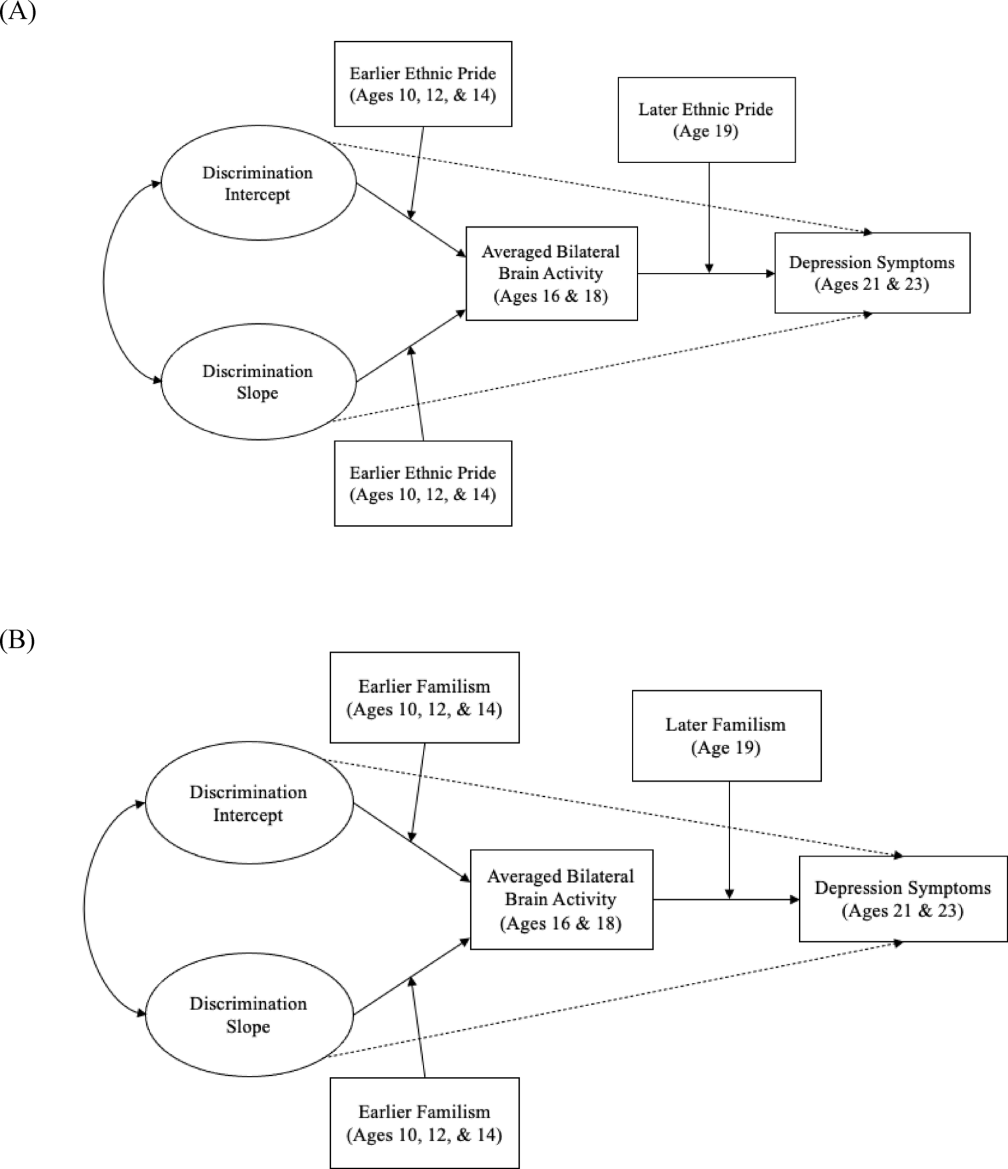
Growth curve model tested for moderating effects of cultural factors. A latent basis growth curve model was used to test longitudinal associations between earlier perceived discrimination, averaged brain activity in later adolescence, and depression symptoms in young adulthood, with (**A**) ethnic pride and (**B**) familism as moderating variables.

The present study examines (1) how perceived discrimination earlier in adolescence (ages 10–14) is associated with depression symptoms in young adulthood (ages 21 and 23; Fig. 1), (2) whether exclusion-related neural activity in later adolescence (ages 16 and 18) mediates this association (Fig. 2), and (3) how cultural factors, in both early and later adolescence, moderate associations between perceived discrimination, exclusion-related neural activity, and depression symptoms (Fig. 3), in a sample of Mexican–American youth studied from adolescence to young adulthood.

To our knowledge, the field lacks research examining the effects of discrimination across the formative years of adolescence on depression outcomes in young adulthood for Latino youth. For this reason, we chose to focus on depression outcomes in young adulthood because not only is this stage of development marked by continued brain maturation, it is also a stage of life that is characterized by distinct experiences that differ from those of early or later adolescents, yet do not fully align with experiences of adulthood^57^. During this time, for instance, young adults further shape their identities, navigate instability in relationships (e.g., work, romantic, home life), and are increasingly focused on self-development and envisioning their future life paths^57^, which makes examining outcomes at this stage a unique window of opportunity to understand what mechanisms in adolescence influence divergent trajectories in young adulthood.

Guided by previous research, we developed three hypotheses. First, we expected that perceived discrimination in earlier adolescence would be positively associated with young adult depression symptoms. Second, we expected that exclusion-related brain function in mid-late adolescence would mediate associations between earlier perceived discrimination and depression symptoms in young adulthood. Lastly, we expected that higher levels of ethnic pride and familism in early^43,58^ and later adolescence^59^ would reduce the effect of perceived discrimination on exclusion-related brain function and reduce the effect of exclusion-related brain function on depression symptoms.

Considering that prior research has found poor test–retest reliability of fMRI measures^60,61^, Elliott and colleagues^62^ have suggested that task-fMRI reliability may be improved with the collection of data at more time points, per individual. For this reason, we focused on three exclusion-related brain regions across two time points (ages 16 and 18) to obtain a more stable estimate of fMRI signal as well as to maximize sample size and increase the reliability of estimates. Regions of interest included the dACC, sgACC, and AI—because (1) social exclusion is closely associated with activity in the dACC and sgACC, two areas involved in processing pain^25,26^, and discrimination is a socially painful experience, (2) activity in the dACC, sgACC, and AI play important roles during social exclusion^33,63^ and have been associated with discrimination^30,64^, and (3) neural function of the dACC, sgACC, and AI during social exclusion are associated with depression^33^. In addition, a review of prior research on neurobiological mediators of discrimination highlighted ACC activity to social exclusion as one of the key neural pathways that may link discrimination to mental health problems^65^. Notably, this literature review also highlighted other potential neural pathways including the salience network and prefrontal cortex, brain networks and regions that are involved in emotion processing and regulation and connected to the ACC, which should be investigated in future research, although they were beyond the scope of the current paper.

## Methods

### Participants

Participants were from the longitudinal California Families Project (CFP). The CFP is an ongoing multiple decade, longitudinal study of 674 Mexican–American youth and their families. Participants from single- and two-parent families were first enrolled into the CFP when they were in fifth grade (mean age = 10.8 years) and subsequent follow-up assessments were conducted annually from age 10 to 19 and then biennially at ages 21 and 23. Participants were selected at random from school rosters in the Woodland and Sacramento, CA school districts (during the 2006–2007 and 2007–2008 school year) and recruited through telephone or, when there was no phone number listed, by a recruiter who went to their home. Participants went through a pre-screening process to determine Mexican-origin ancestry, that the child was living with his/her biological mother, and the father in two-parent families had to be the child’s biological father. The parent CFP study from which these data were drawn included only participants living with biological parents due to the broader aims and goals of the CFP study to examine community, family, and individual characteristics that promote children’s development with a focus on two-parent and single-parent families in order to better focus on these specific parent–child relationships. All procedures were approved by the UC Davis IRB and all methods were performed in accordance with the relevant guidelines and regulations; parents provided informed consent and adolescents provided informed assent before beginning study procedures, and once the adolescent participants turned 18 they provided informed consent. Details on retention and attrition analyses for the study variables of interest can be found in supplementary materials (Appendix S1, Tables S1 & S2).

At around age 16, data collection began for a neuroimaging sub-study in which participants completed a social exclusion task (Cyberball) during an fMRI scan. For further details about the sub-study see Beard et al.^27^; Schriber et al.^30^; and Weissman et al.^66^. The depression risk sub-study recruited adolescents from the parent sample whose self-reported depression symptoms in 9th grade (age 14 to 15) were high (but not clinically diagnosed as depressed)—as assessed by the Diagnostic Interview Schedule for Children-IV^67^ as well as two subscales of the Mood and Anxiety Symptom Questionnaire^68^—General Distress and Anhedonic Depression. To achieve variability across adolescents who were recruited for the sub-study, the sample included adolescents with symptom scores above the median on all three measures of depression (N = 43), on two measures (N = 64), on one measure (N = 68), and those at or below the median on all three measures (N = 54), ensuring a range of symptom variability. The median scores for these measures at the time of recruitment were: MASQ General Distress = 1.00 (*M* = 1.32), MASQ Anhedonic Depression = 2.25 (*M* = 2.35), and C-DISC MDD symptom count = 3.00 (*M* = 4.09). No participants met the diagnostic criteria for MDD at the time of recruitment. Neuroimaging data using the Cyberball task involving these participants have been published in previous research^27,30^. Stein et al.^2^ has also published work using the CFP data to examine longitudinal associations between discrimination and depression from 5th to 12th grade but with the full sample of youth and using a different longitudinal model.

Neuroimaging data were collected at two time points for the depression sub-study, age 16–17 for time 1 and age 18 for time 2. Schriber et al.^30^ has previously reported on T1 data from the depression risk sub-study for the Cyberball task. Of the 229 participants who enrolled in the neuroimaging sub-study at T1, 33 were excluded—10 were excluded due to an MRI contraindication determined at the visit, 2 were excluded due to an equipment malfunction, and 21 participants were excluded for excessive head motion. The resulting total sample for T1 analyses was 196 adolescents (51% female; *M* age at MRI scan = 16.67 years, *SD* = 0.43). At T2, 162 participants completed a scan visit, with 11 then excluded for data quality control issues. Thus, for T2, the final sample size comprised 151 participants (43.1% female; *M* age at MRI scan = 18.60 years, *SD* = 0.42). See supplementary Figure S1 for a flowchart on exclusion of participants at both T1 and T2.

For the present study, to maximize the sample size we included participants if they had fMRI data at either T1 or T2 and averaged data between T1 and T2 for participants who had data at both time points for main analyses. This resulted in a sample size of 196 participants (48.3% female; *M* age at MRI scan = 17.63 years, *SD* = 0.43, range = 15.85–19.88 years). Participant characteristics are reported in Table 1 and bivariate correlations are reported in Table 2. We also conducted post hoc sensitivity analyses for participants that had fMRI data at both T1 and T2 (*n* = 146) and post hoc sensitivity analyses to examine how changes in brain function across T1 and T2 were associated with depression symptoms. The latter sensitivity analyses were motivated by previous research that found cortical development continues throughout late adolescence^69^.

**Table 1:** Descriptive statistics.

Total sample (*n* = 196)				
	Mean	*SD*	Min	Max
Adolescent age (Time 1 fMRI scan)	16.67	0.43	15.85	18.02
Adolescent age (Time 2 fMRI scan)	18.60	0.42	17.83	19.88
Experiences of discrimination (Age 10)	1.26	0.43	1	3.25
Experiences of discrimination (Age 11)	1.19	0.49	1	4
Experiences of discrimination (Age 12)	1.06	0.17	1	2.25
Experiences of discrimination (Age 13)	1.08	0.27	1	3.75
Experiences of discrimination (Age 14)	1.09	0.54	1	2
Depression symptoms (Age 14)	2.00	0.54	1	4
Depression symptoms (Ages 21 & 23)	2.18	0.54	1.46	4.15
Early ethnic pride (Ages 10, 12, 14)	3.40	0.38	2.17	4.67
Later ethnic pride (Age 19)	3.20	0.71	1	4
Early familism (Ages 10, 12, 14)	3.53	0.31	2.33	5
Later familism (Age 19)	3.36	0.61	1	4
Family income (Average across ages 10–14)	7.23	4.56	1	20
Mother education level	9.48	3.40	1	16
Father education level	8.97	3.43	1	16

SD ,  Standard deviation; Min , Minimum; Max , Maximum. Family income was coded as an ordinal variable, with higher values indicating higher income brackets (e.g., 1 = Less than $5,000; 20 = $95,000 or more). For reference, a score of 7 corresponds to $30,001–$35,000 and 8 to $35,001–$40,000. The mean income score was 7.23, suggesting an average income range between approximately $30,001 and $40,000. Parent education level was also coded ordinally, reflecting years of schooling (e.g., 1 = 1st grade; 16 = 4 + years of college, BA/BS degree).

**Table 2:** Bivariate correlations.

	Ethnic disc (age 10)	Ethnic disc (age 11)	Ethnic disc (age 12)	Ethnic disc (age 13)	Ethnic disc (age 14)	Average bilateral sgACC activity	Dep sx (age 21 & 23)	Early ethnic pride (ages 10,12,14)	Early familism (ages 10,12,14)	Later ethnic pride (age 19)	Later familism (age 19)	Sex	Dep sx (age 14)
Ethnic disc (age 10)	1												
Ethnic disc (age 11)	.18**	1											
Ethnic disc (age 12)	.10	.05	1										
Ethnic disc (age 13)	.01	− .03	.26**	1									
Ethnic disc (age 14)	.11	− .01	.23**	.15**	1								
Average bilateral sgACC activity	.03	− .24**	.09	− .03	.06	1							
Dep sx (age 21 & 23)	− .05	.02	.02	− .11	.17*	.07	1						
Early ethnic pride (ages 10,12,14)	− .16*	− .12	− .01	.10	− .06	.04	.11	1					
Early familism (ages 10,12,14)	.03	− .18*	− .10	.10	− .09	− .02	− .10	.34	1				
Later ethnic pride (age 19)	.09	− .03	.03	.08	− .01	.15*	− .08	.37**	.17*	1			
Later familism (age 19)	.12	− .10	− .01	.02	.00	.06	− .14	.16*	.31**	.56**	1		
Sex	− .15*	.08	− .06	− .05	− .10	-.001	− .23**	− .10	− .03	− .34**	− .10	1	
Dep sx (age 14)	.08	.07	.04	.00	.22	.06	.30**	− .17*	− .20**	− .09	− .26**	− .19**	1

*Correlation is significant at the 0.05 level (2-tailed). ** Correlation is significant at the 0.01 level (2-tailed). Disc = Discrimination; Dep Sx = Depression Symptoms; Sex was coded as 1 = male and 0 = female.

### Measures

#### Self-report measure: adolescent’s perceptions of discrimination questionnaire

At ages 10, 11, 12, 13, and 14, participants completed the Adolescent’s Perceptions of Discrimination (DSCR) Questionnaire^70,71^ to assess adolescents’ perceptions of, and personal experiences with, discrimination. All questions were rated on a scale ranging from (1) “Not at all true” or “Almost never/Never” to (4) “Very true” or “Almost always/Always”. For the purposes of this study, we focused on the 5 items that pertain to personal experiences of discrimination (i.e., “How often have kids at school excluded you from their activities, like not inviting you to go out with them, not inviting you to their houses, or not letting you join their games, because you are [Mexican/Mexican–American]?”), which demonstrated low to good reliability ($\Omega_{Wave 1 - 5} = .51 - 82)$ but had relatively low variability across participants (see means and standard deviations in Table 1). The five items were averaged to calculate a mean score of personal experiences of discrimination at each age.

#### Self-report measure: Mexican–American ethnic pride scale

At ages 10, 12, 14, and 19, participants completed the Mexican American Ethnic Pride Scale, which is adapted from the Multigroup Ethnic-identity scale^72^. The Ethnic Pride scale includes 8 items that assess identification with Mexican/Mexican–American culture, including how good or proud one feels about their culture and how much one participates in Mexican/Mexican–American cultural traditions (i.e., “You are happy that you are [Mexican/Mexican American]”, “You feel good about your cultural or ethnic background”). All questions were rated on a scale ranging from “Not at all true” (1) to “Very true” (4). The 8 items were averaged to derive a mean score for earlier levels of ethnic pride across ages 10, 12, and 14 for use as a moderator in analyses (see Fig. 3) and demonstrated sufficient reliability in this sample (${\Omega }_{Waves 1, 3, \& 5}=.77-.83)$. Ethnic pride at age 19 also demonstrated good reliability (${\Omega }_{Wave 10}=.90$).

#### Self-report measure: Mexican–American cultural values familism scale

At ages 10, 12, 14, and 19, participants completed the Mexican American Cultural Values Familism Scale (MACVS)^73^. The MACVS includes three subscales measuring aspects of familism: familism support (i.e., responsibility to maintain close relationships to the family, such as maintaining close relationships with extended family as well as examining the importance of getting together with extended family for important celebrations and holidays), familism obligations (i.e., obligation to the family such as, sharing your room with relatives if they need a place to stay, taking care of siblings, and/or being a role model for younger siblings), and familism referent (i.e., importance of how your actions act as a reflection upon the family such as expectations to behave well and thinking about the family when making important decisions). All questions were rated on a scale ranging from “Not at all” (1) to “Completely” (5). The familism subscales were averaged to derive a mean score for earlier levels of familism at ages 10, 12, and 14 for use as a moderator in analyses (see Fig. 3) and demonstrated good reliability (${\Omega }_{Waves \text{1,3},\&5}=.83-.86)$. Familism at age 19 also demonstrated good reliability (${\Omega }_{Wave 10}=.92$).

#### Self-report measure: mini-mood and anxiety symptom questionnaire

At ages 14, and 21 and 23, participants completed the Mini-Mood and Anxiety Symptom Questionnaire (MASQ)^68^. To assess depression symptoms in young adulthood (averaged across ages 21 and 23), and as a covariate in early adolescence (age 14), we averaged the general distress and anhedonic depression subscales. The anhedonic depression subscale items assessed feelings of happiness, joy, energy levels, and interest in doing activities that are fun (i.e., “How much have you felt like you had a lot of energy?”). This subscale was reverse-coded so that higher values represent higher levels of anhedonia. The anhedonic depression subscale items also demonstrated good reliability (${\Omega }_{age 14 and 21 \& 23}=.88\text{ and }.86)$. The general distress subscale included questions that assessed feelings of hopelessness, discouragement, failure, and low self-worth (i.e., “How much have you felt discouraged? Felt hopeless?”). The general distress subscale items demonstrated good reliability (${\Omega }_{age 14 and 21 \& 23}=.90\text{ and }.93)$. All items were rated on a scale ranging from “Not at all” (1) to “Extremely” (5).

#### Cyberball social exclusion fMRI task

The Cyberball task is a measure of social exclusion in which participants are either included or excluded from a simulated ball-tossing game^22^. During the task, participants play 12 rounds of the simulated ball-tossing game. The game is comprised of three players (two simulated players and the participant) and includes six rounds each of Inclusion and Exclusion. The Cyberball game is always presented in the following order of rounds: Inclusion, Exclusion, Inclusion, Inclusion, Exclusion, Inclusion, Exclusion, Inclusion, Exclusion, Exclusion, Exclusion, and Inclusion. Each round lasts 36 s. During the Inclusion round, simulated players are equally likely to throw the ball to the participant or one another. During the Exclusion round, the simulated players stop throwing the ball to the participant and instead, continue throwing the ball to one another. When the game begins participants are shown a fixation point (4 s), then a “Begin Match!” notification (2 s), followed by 10–11 simulated ball tosses of game play (22–23 s) that includes all relevant players’ ball tosses, and then a short reloading screen (7–8 s). Instructions (8 s) are also provided at the start of the Cyberball task and the task concludes with a “Thank you!” (3 s) to signify completion of the task. The Cyberball task is implemented in one run and lasts 7 min 23 s.

### Cyberball fMRI data analysis

#### BOLD fMRI data acquisition, preprocessing, quality assurance, and first-level analysis

Imaging data were collected using a Siemens 3 T Tim Trio scanner with a 32-channel head coil. Adolescents were given extensive instructions to decrease head motion, which was also limited with foam padding and surgical tape. Following sagittal localization and a manual shim procedure, whole-brain BOLD functional T2*-weighted echo-planar images (EPI) for each brain volume were acquired and consisted of a 64 × 64 matrix (repetition time [TR] = 2000 ms; echo time [TE] = 27 ms; flip angle = 80°; field of view [FOV]) = 224 mm; 3.5 × 3.5 × 3.5 mm voxel size, slice thickness = 3.5 mm).

Preprocessing was conducted using Analysis of Functional NeuroImaging (AFNI) software version 16.2.09 (www.afni.nimh.nih.gov). For each subject, preprocessing of EPI data included slice time correction, motion correction, reslicing to a 2 mm isotropic voxel, spatial smoothing (4 mm full-width half-maximum Gaussian kernel), and normalization of BOLD signal intensity to percentage signal change using each subject’s voxel-wise time series mean as a baseline. Each participant’s functional dataset was co-registered with their structural images and normalized to Montreal Neurological Institute stereotaxic space using a two-stage registration in FSL. Alignment of functional data to structural images was visually confirmed for all participants. AFNI was then used to censor volumes with head motion greater than 1 mm from the previous volume. Quality control criteria required the exclusion of participants with ≥ 10% volumes censored for head movement, if ≥ 20% of repetition times (TR) were outliers from the previous TR, and with poor image quality based on visual inspection.

For first-level processing, Cyberball was modeled as a block design consisting of Inclusion or Exclusion conditions and the following regressors were modeled: Inclusion, Exclusion, instructions, “Begin match!” screen, and button presses to control for motor activity. Exclusion and Inclusion were modeled as boxcar functions with an amplitude of 1 using AFNI’s duration modulation (dmBLOCK) to account for duration variability due to reaction time differences. Motion regressors, that were not of interest, were modeled using gamma functions. All were convolved with a canonical hemodynamic response function. Further information on fMRI data processing and analyses can be found in Schriber et al.^30^. Linear contrasts were calculated that compared blood-oxygen level–dependent (BOLD) responses in the Exclusion > Baseline condition and Inclusion > Baseline condition for each participant. Due to our focus on exclusion-related neural activity, we focused on the Exclusion > Baseline condition for analyses. Additionally, we focused on the contrast of Exclusion > Baseline rather than Exclusion > Inclusion because prior research indicates that contrasts relative to baseline are more stable than contrasts comparing two experimental conditions^75^.

For second-level processing, a structural region-of-interest (ROI) approach was used to assess BOLD responses to Exclusion > Baseline within the sgACC, dACC, and AI, selected a priori based on previous publications using this same sample (see Supplementary Appendix S2 for ROI definition)^30^. Neural activity at both time points was processed with the same processing pipeline and activity from both time points was extracted using structural ROIs to standardize the extraction of activity across the time points. Contrast values were extracted from bilateral structural ROIs at each time point separately, and then averaged, before submitting them for further statistical analyses. Given that averaging brain data across two time points may obscure potential developmental changes occurring across these time points, we also conducted a supplementary analysis examining whether change in activity across the time points predicted depression symptoms (see Statistical Analyses section for further details).

Intraclass correlation coefficients (ICC) between the two time points of extracted Exclusion > Baseline contrast values for the three ROIs were calculated to determine test–retest reliability of the ROIs across time. ICCs were examined to assess the stability of neural activation from T1 to T2 during the Cyberball task, determining if neural activation was strongly correlated across these time points. Because we planned to average neural activity, nonsignificant ICCs would suggest that neural activation may not have been stable across the two time points. If this was the case, we did not average neural activity for any measurements with nonsignificant ICCs from T1 to T2. After running the ICCs, only the bilateral sgACC had a statistically significant ICC (ICC = 0.31, *p* = 0.02; see Supplementary Appendix S3 for additional ICCs). Therefore, we only included sgACC activity during Exclusion vs. Baseline in analyses, as it was the only region showing stable neural activation across the two time points. All self-report measures and bilateral sgACC contrast values were imported into R (Version 4.3.0)^74^ for data analysis.

### Statistical analyses

Perceived discrimination trajectories were estimated using latent growth curve (LGC) modeling and analyses were run using the package *lavaan* in R (*Version 0.6.15*)^76^. Missingness was accounted for using full information maximum likelihood (FIML) estimation. To allow for the possibility that perceived discrimination may exhibit either linear or non-linear change over time, we compared a series of growth models (linear, latent basis, quadratic) to identify the specific growth trajectory that best represents the observed data and to allow for different possible types of change in this construct. The latent basis model was the best-fitting model, while the linear and quadratic growth curve models had poor fit (see Table S3). This suggests that discrimination did not exhibit linear or quadratic growth, but was best captured by non-linear change over time. For this reason, we determined that a latent basis growth trajectory was most suitable for our data. See Supplementary Appendix S4 for guidelines used to determine good model fit. Please also see Supplementary Appendix S5 for a description of how we corrected analyses for multiple comparisons. See also Figure S2 for a flowchart outlining our analytical steps.

Our first hypothesis was that perceived discrimination across multiple years of adolescence would be associated with depression in young adulthood. To test this hypothesis, we ran a latent basis growth curve model that examined how the level and slope of the perceived discrimination trajectory were associated with depression in young adulthood (See Fig. 1 for model). Overall fit measures suggested that the fit of the model was acceptable (Table 3). The intercept and slope variances for perceived discrimination are reported in supplementary materials Table S4. We also ran post hoc sensitivity analyses for two separate latent basis growth curve models that examined how the level and slope of perceived discrimination were associated with anhedonic depression and general distress in young adulthood (results for these analyses are reported in Appendix S6, Figure S3).

**Table 3:** Fit statistics for latent growth curve models examining associations between discrimination, bilateral sgACC brain activity, cultural factors, and depression symptoms.

Model	CFI	TLI	RMSEA	χ^2^(df)	*p*
Figure 1	1.00	1.00	0.00	32.57 (4)	< 0.001
Figure 2	1.00	1.31	0.00	25.02 (9)	0.003
Figure 3 (A)	1.00	1.23	0.06	30.80 (15)	0.009
Figure 3 (B)	0.82	0.31	0.07	32.35 (15)	0.006

CFI = comparative fit index; TLI = Tucker Lewis Index; RMSEA = root-mean-square-error of approximation.

Our second hypothesis was that exclusion-related brain activity would mediate associations between perceived discrimination and depression. To test this, we examined the association between the perceived discrimination trajectory (across ages 10–14) and exclusion-related brain function (averaged across 16 and 18), and the association between exclusion-related brain function and depression (averaged across ages 21 and 23) (See Fig. 2 for model). Overall fit measures suggested that the fit of the model was acceptable (Table 3). We tested for mediation by bootstrapping confidence intervals. We also repeated the mediation analyses separately for anhedonic depression and general distress (results are reported in Appendix S6, Figure S4). Lastly, we conducted sensitivity analyses to examine associations between changes in sgACC activity across T1 and T2 as a predictor of young adult depression symptoms, using the same methods as described above (results are reported in the Supplement in Figure S5).

Our third hypothesis was that cultural factors (ethnic pride and familism) would moderate associations between the perceived discrimination trajectory (level and slope) and exclusion-related brain function, as well as moderate associations between exclusion-related brain function and depression (See Fig. 3 for model). To test our hypotheses, we ran moderation analyses. In this model, we created a variable for earlier levels of ethnic pride (averaged across ages 10, 12, and 14) and included this variable as a moderator of the paths from the intercept and slope of perceived discrimination to bilateral sgACC activity (averaged across age 16 and 18), and then simultaneously tested age 19 levels of ethnic pride as a moderator on the path from bilateral sgACC activity (averaged across age 16 and 18) and depression (averaged across ages 21 and 23). We planned to probe significant interactions with simple slopes analysis. In the fourth model, the same approach from above was used for assessing the moderating effect of familism on associations between earlier perceived discrimination, brain activity in later adolescence, and depression in young adulthood. Similar to what was done for ethnic pride, we created a variable for earlier familism (averaged across ages 10, 12, and 14) and included this variable as a moderator on the paths from the intercept and slope of earlier perceived discrimination to bilateral sgACC activity (averaged across age 16 and 18), and then simultaneously tested age 19 levels of familism as a moderator of the path from bilateral sgACC activity (averaged across age 16 and 18) to depression. Overall fit measures suggested that the fit of these models was acceptable (Table 3). We repeated these analyses separately for anhedonic depression and general distress (these results are reported in Appendix S6, Figure S6).

Lastly, we conducted sensitivity analyses on participants who had brain data available at both time points using the same methods as above (results of these analyses are reported in Appendix S7, Figure S7).

### Covariates

We included adolescent sex as a covariate in all growth curve analyses to control for any covariance of sex with perceived discrimination, depression, and brain activity, given evidence that women are more likely to be diagnosed with depression than men^77,^ and because Latino men and women have different experiences with perceived discrimination^78^. In all the models, we also controlled for levels of depression at age 14 to examine how perceived discrimination and neural activity predict relative changes in depression symptoms while controlling for earlier levels of depression symptoms.

## Results

### Bivariate correlations

With the exception of a negative correlation between perceived discrimination (age 11) and average bilateral sgACC activity (across ages 16 & 18; *r* = − 0.24, *p* < 0.01, Table 2), and a positive correlation between perceived discrimination (age 14) and depression (ages 21 and 23; *r* = 0.15, *p* < 0.05; Table 2), there were no other significant correlations between earlier experiences of perceived discrimination, average bilateral sgACC activity, and depression (all *p*’s > 0.05).

### Association between earlier perceived discrimination and young adult depression symptoms

Contrary to expectations, the intercept and slope of perceived discrimination across early adolescence were not significantly associated with depression symptoms in young adulthood (all *p’s* > 0.05, Figure S3).

### Mediating effect of exclusion-related brain activity on associations between earlier perceived discrimination and depression symptoms in young adulthood

Given our hypothesized mediation effect, we expected to find that perceived discrimination is associated with neural activity and neural activity is associated with depression. However, we did not find support for either pathway. Specifically, the intercept and slope of perceived discrimination across early adolescence were not significantly associated with bilateral sgACC activity in later adolescence, and bilateral sgACC activity was not significantly associated with young adult depression (all *p’s* > 0.05, Figure S4). Exploratory analyses examining change in bilateral sgACC activity from T1 to T2 predicting depression were also nonsignificant (*p* > 0.05, Figure S5).

### Moderating effect of cultural factors on associations between earlier perceived discrimination, exclusion-related brain activity, and depression symptoms

**Fig. 4:**
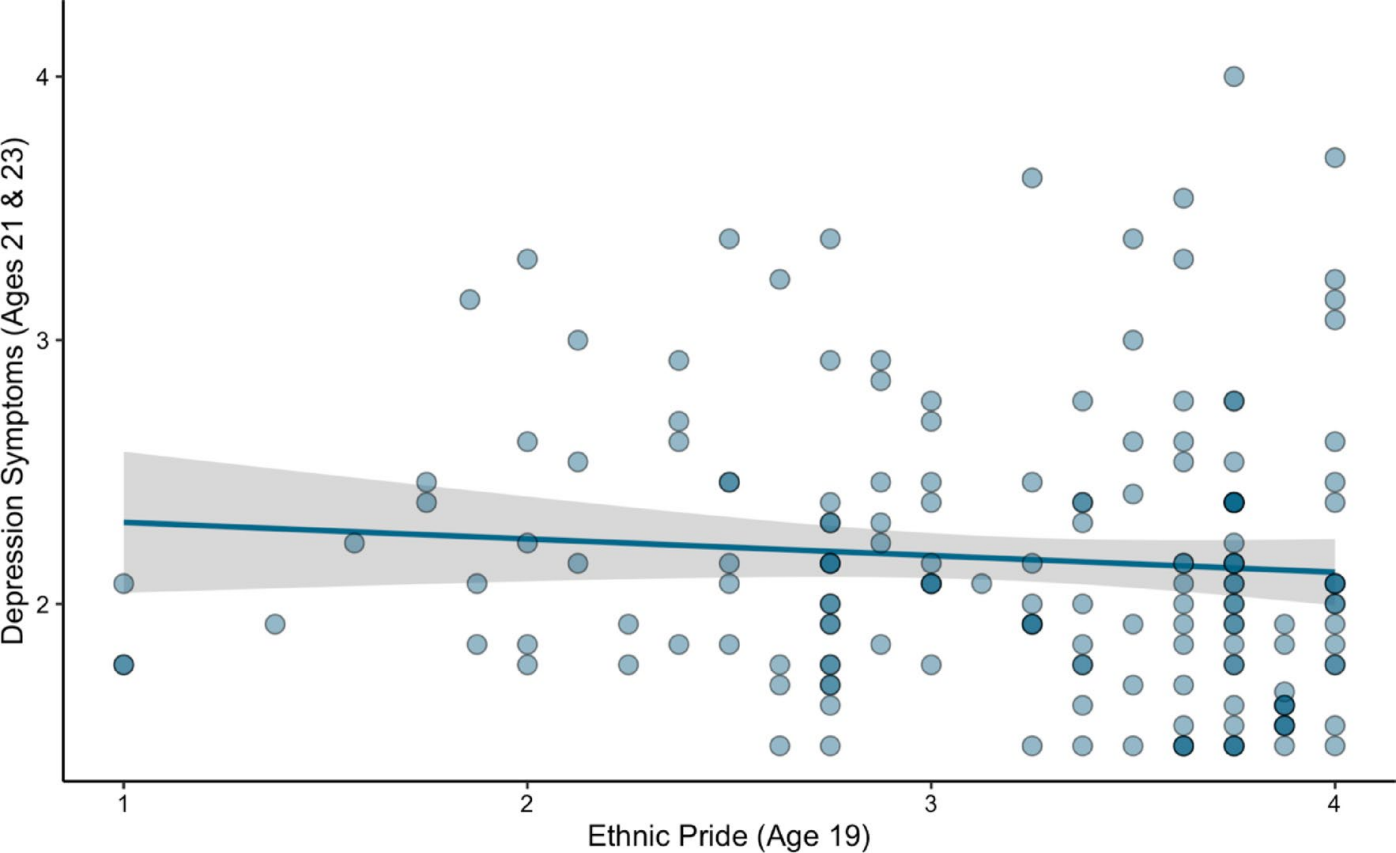
Association between ethnic pride and young adult depression symptoms. Regression plot demonstrates association between later ethnic pride on depression symptoms.

In partial support of our hypotheses, ethnic pride had a significant main effect on depression. Specifically, higher ethnic pride in later adolescence was associated with lower depression in young adulthood ($\beta$ = − 0.17, *SE* = 0.06, *p* = 0.007; Table 4, Fig. 4). We also found that earlier ethnic pride was positively associated with later depression ($\beta$ = 0.38, *SE* = 0.11, *p* = 0.001, Table 4) but this significant finding for earlier ethnic pride emerged when it was included as a covariate on the pathway examining later ethnic pride as a moderator of associations between brain activity and depression.

**Table 4:** Regression model results examining moderating effect of ethnic pride on associations between earlier experiences of discrimination, later adolescent bilateral sgACC brain activity, and young adult depression symptoms.

	*β*	*SE*	95% CI	*p*
*Path 1: Outcome variable—Bilateral sgACC activity*				
Discrimination (intercept)	− 0.08	0.52	(− 0.21 to 0.40)	0.88
Discrimination (slope)	0.17	0.61	(− 0.18 to 0.44)	0.78
Early ethnic pride	− 0.42	1.09	(− 0.12 to 0.18)	0.70
Discrimination (intercept) * Early ethnic pride	0.39	1.00	(− 0.20 to 0.61)	0.70
Discrimination (slope) * Early Ethnic Pride	0.28	1.06	(− 0.23 to 0.53)	0.79
Sex	− 0.01	0.03	(− 0.30 to 0.28)	0.85
*Path 2: Outcome variable—Depression symptoms*				
Averaged bilateral sgACC brain activity	− 0.09	1.05	(− 2.16 to 1.97)	0.93
Later ethnic pride	− 0.17	0.06	(− 0.29 to − 0.05)	0.007
Averaged bilateral sgACC brain activity * later ethnic pride	0.09	0.32	(− 0.54 to 0.71)	0.79
Sex	− 0.21	0.08	(− 0.37 to − 0.05)	0.01
Early ethnic pride	0.38	0.11	(0.16 to 0.59)	0.001
Earlier depression symptoms	0.29	0.07	(0.15 to 0.43)	< 0.001

SE = Standard errors, CI = the 95% confidence intervals, β = standardized regression coefficients. Significant values (*p* < .05) are bolded.

We did not find evidence for any of the hypothesized moderating effects of cultural factors. Specifically, earlier ethnic pride did not moderate associations between earlier perceived discrimination and bilateral sgACC brain activity, and later ethnic pride did not moderate associations between bilateral sgACC brain activity and depression (all *p’s* > 0.05, Table 4). Similarly, earlier familism did not moderate associations between earlier perceived discrimination and bilateral sgACC brain activity, and later familism did not moderate associations between bilateral sgACC brain activity and depression (all *p’s* > 0.05, Figure S6). When examining anhedonic depression and general distress separately, later ethnic pride predicted lower anhedonic depression symptoms in young adulthood, but later ethnic pride and familism were not associated with lower young adult general distress (Figure S6).

## Discussion

The mechanisms of risk and resilience to depression are important to understand because depression impairs psychosocial functioning^79^ and often develops during adolescence^80^. Latino youth are at particularly high risk for depression^81^, underscoring the need to understand risk and protective factors for depression in this population. The aim of the present study was to investigate longitudinal associations between perceived discrimination, later adolescent exclusion-related brain activity, and young adult depression in a sample of Mexican–American youth. This study also examined the moderating effects of two cultural factors, ethnic pride and familism.

Our first hypothesis that perceived discrimination in early adolescence would be associated with young adult depression was not supported, despite prior evidence suggesting that discrimination impacts depression outcomes for Latino youth^2,17^. There are several reasons why we may not have found significant effects for this hypotheses. First, low values and lack of variability in the perceived discrimination measure may have led to weaker effects for all of our models. This could be because adolescents growing up in an ethnically and racially diverse region like Northern California may experience lower rates of discrimination than in other regions of the country. Or, perhaps adolescents’ experiences of discrimination in early adolescence are not well captured by our discrimination measure. Further, other buffers in youths’ lives such as social support (i.e., peers, family, school context) could have ameliorated the expected effect. Lastly, there was a long time interval between discrimination and depression and it is possible that experiences of discrimination affect depression levels one or two years later but not all the way from early adolescence (ages 10–14) to young adulthood (ages 21 & 23).

Interestingly, we also did not find support for our second hypothesis that exclusion-related neural activity would mediate longitudinal associations between earlier perceived discrimination and young adult depression. Very few studies have examined associations between perceived discrimination and exclusion-related neural activity^5,30^. We may not have seen significant effects in our study due to low levels of discrimination, the gap in time between discrimination, brain, and depression measures, as well as a smaller sample size for the growth curve analyses that were performed. An additional reason for why we may not have seen significant effects is that the Cyberball task may not adequately capture social processing related to discrimination. In future work, a more ecologically valid neuroimaging task could be used to better capture neural activity related to racial discrimination. For example, the nuanced affectively salient and social aspects of this experience could be measured via an fMRI paradigm that involves more “real-life” characters (e.g., out-group exclusion) and/or approximates discrimination experiences based on race rather than game-playing as was done here. For instance, one ERP study examined social exclusion within the context of ingroup and outgroup status and found ERP activation in brain regions similar to those recruited for the traditional Cyberball task^82^. We discuss implications of the traditional Cyberball task further in the limitations section.

In partial support of our third hypothesis, we found that ethnic pride at age 19 appears to predict lower depression in young adulthood. We also found that earlier ethnic pride predicted young adult depression but in an opposite direction, which could suggest that stronger ethnic pride in early adolescence, when youth are actively exploring their identities and when experiences of rejection are particularly salient, may lead Latino adolescents to perceive and react more strongly to experiences of discrimination based on their ethnic group identity. For instance, increases in ethnic pride might lead to heightened awareness of discrimination and the social challenges associated with it, potentially increasing the risk of depressive symptoms. However, we emphasize that the relationship between ethnic pride and depression is complex and can vary depending on individual experiences and environmental factors. This suggests that the developmental stage when ethnic pride is assessed may be important for determining how it relates to young adult depression symptoms, with ethnic pride in late adolescence appearing to predict lower depression in young adulthood but perhaps not so in earlier adolescence. However, we did not find support for our hypotheses that cultural factors moderated associations between perceived discrimination in earlier adolescence, exclusion-related brain activity in later adolescence, and young adult depression. It is possible that we only found partial support for our hypotheses because, as mentioned previously, we did not have enough variability in the perceived discrimination measure and the Cyberball task may not accurately capture social exclusion related to discrimination. We also only examined brain activity in this study, but examining brain connectivity could reveal neural networks that mediate associations between experiences of discrimination, brain function, cultural factors, and depression symptoms. In future work, other model selections may reveal patterns not reported here. For example, an alternate approach could be to model depression longitudinally and examine discrimination as a time-varying predictor.

Nonetheless, our finding that ethnic pride is negatively associated with depression appears to align with previous research suggesting that ethnic-racial socialization may mitigate negative mental health outcomes^37^. And when examining anhedonic depression separately, later ethnic pride predicted lower anhedonic depression symptoms. Previous research has found that ethnic pride protects against negative psychological outcomes in Latino youth^16,40,41,45^. Despite the breadth of existing literature on associations between cultural factors and depression in Latino youth, a large portion of this research has been cross-sectional^16^, ^41^ or focused solely on the developmental period of adolescence^83^, with relatively limited research examining the effects of cultural factors in later adolescence and their associations with depression in young adulthood. The findings from this study therefore extend previous findings by highlighting the importance of ethnic pride in later adolescence which in turn, may help compensate against the development of depression as youth make the transition to young adulthood—a time when youth are starting to make important decisions about their futures, while simultaneously learning how to navigate the world more autonomously.

In addition to the methodological limitations already noted above, it is important to note several other limitations. First, two of the regions of interest for neural activity (bilateral AI and bilateral dACC) had non-significant test–retest reliability across T1 and T2, which limited our ability to examine brain regions known to be associated with social exclusion. Nevertheless, we did observe significant test–retest reliability for one of our selected ROIs, and by averaging across the two time points were able to examine a more stable estimate of brain activity for this region. We believe the limitation of poor test–retest reliability for neural activity to social exclusion may hold for many other studies of neural activity and depression because most neuroimaging studies examine one time point of neural activity and therefore are unable to examine test–retest reliability, indicating the need for the design of tasks that show more robust test–retest reliability over time. It could also be the case that brain activity with low ICC values may be thought of more as state-like markers that fluctuate over time, rather than trait-like markers that can predict future outcomes^84^. Second, the current study does not account for the intersectional influence of variables (i.e., gender, cultural factors, acculturative stress, social class) on brain function across multiple groups of minoritized youth. For instance, associations between racial discrimination, neural function, and depression could manifest differently based on multiple forms of marginalized identities one might hold, and the more marginalized identities an individual has, the more complex the intersectional influence of these variables will be. In future work it will therefore be imperative to leverage larger datasets, such as the Adolescent Brain Cognitive Development longitudinal study^85^ in order to capture this complex intersection of developmental experiences across diverse samples of adolescents. Lastly, when studying the neural correlates of brain development, an integration of cultural neuroscience can be utilized to more accurately capture the brain mechanisms recruited for a given function^86^. For example, existing studies examining Latino and White youth suggest that brain function could vary based on cultural values^87^ and because the Cyberball task does not take cultural variability into account, future work would benefit from tailoring the Cyberball task to more accurately capture experiences of discrimination. By accounting for these factors in future work with adolescents, we can begin to understand the effects of discrimination as an experience of adversity and the effects discrimination has on neurobiology and psychosocial functioning. Even further, given the effects of ethnic pride on lower depression symptoms, in future work, it may also be important to examine cultural factors as neural correlates or predictors of brain processes.

The present study begins to elucidate gaps in the literature by examining associations among discrimination, brain activity during social exclusion, cultural factors, and depression, and advancing our understanding of how experiences of social exclusion, as it relates to discrimination, affect depression outcomes for Latino youth. Our main finding from this study highlights the role of positive ethnic pride in later adolescence, but not in earlier adolescence, as a predictor of lower depression symptoms in young adulthood. These findings can help to guide the tailoring of interventions for Mexican–American young adults facing specific types of adversity using equitable strengths-based approaches that capitalize on cultural factors to eliminate mental health disparities.

## Supplementary Information

Below is the link to the electronic supplementary material.

Supplementary Material 1

## Data Availability

The raw data from this study cannot be shared since participants did not consent to the public sharing of data. However, the data that support the findings of this study are available on request from the corresponding author.
