# Supplementary material for: Identifying neural mediators and cultural moderators of the association between discrimination and depression among Mexican American youth
Source: Sci Rep. 2025 Nov 6;15:38924. doi: 10.1038/s41598-025-22900-8 (PMC12592538; doi:10.1038/s41598-025-22900-8)
Supplement: Supplementary file 1 — Supplementary Material 1 [file 41598_2025_22900_MOESM1_ESM.docx]

**Supplementary Materials**

**Appendix S1.** Participant retention and attrition.

In Table S1 we provide additional details on retention/attrition for the CFP study across each wave of data collection. We also performed attrition analyses using a paired samples t-test to examine whether there were significant differences in the brain, discrimination, depression, ethnic pride, and familism study variables between participants who had data at all waves versus those who were missing data on one or more waves. Attrition analyses indicated that depression symptoms in young adulthood were higher in participants with data at all waves compared to participants missing data at one or more waves. We found no other significant differences between participants who had data at all waves versus those who did not (Table S2).

**Appendix S2.** Definition of sgACC, dACC, and AI ROIs.

The ROI for the bilateral sgACC was created from the right and left Brodmann Area (BA) 25 masks (defined by the Talairach-Tournoux database) and transformed to MNI space using the tta2mni function, then modified to include only areas of BA 25 that were under the genu of the corpus callosum posterior to y = 30, using the whereami function in MNI space, as “cingulate cortex,” within AFNI. Based on prior criteria (Vogt, Berger, & Derbyshire, 2003), the ROI for the bilateral dACC was created using the “cingulate cortex” mask in the MNI database which was modified to use a rostral boundary of y = 32, which has been done previously (Shaffer et al., 2000), and a caudal boundary of y = 0 based on prior studies of social exclusion using this same sample (Schriber et al., 2018). Then, for the bilateral AI, we used AFNI’s whereami function in MNI space as “insula,” and merged the right and left insula masks anterior to the y = 0 plane (Meyer, Williams, & Eisenberger, 2015).

**Appendix S3.** Reporting ICCs for ROIs.

Because we planned to average neural activity across the two time points, we examined each measure of neural activity to see if it had significant test-retest reliability across the two-year interval. The bilateral sgACC for T1 and T2 had a statistically significant ICC of 0.31 (*p* = .02). The ICC for bilateral AI activity for T1 and T2 was 0.12 (*p* = .23). The ICC for bilateral dACC activity for T1 and T2 was -.11 (*p* = .72). Given the low ICC and non-significant p-values for bilateral AI and bilateral dACC, we focused our analyses solely on average brain data for the bilateral sgACC.

**Appendix S4.** Guidelines for determining model fit.

To determine good model fit, we used the chi-square test (χ2), the confirmatory fit index (CFI; Bentler, 1990), and root mean square error of approximation (RMSEA; Steiger, 1990). The χ2 statistic is sensitive to sample size (Bollen, 1989), such that models with larger sample sizes tend to result in a significant χ2 despite having appropriate fit to the data. We therefore chose to evaluate CFI and RMSEA when considering model fit and conducting model comparisons. We evaluated model fit by the CFI, with values above 0.90 indicating good fit, and values between .70 - .80 indicating moderately good fit (Bentler, 1990). For evaluating model fit by the RMSEA, values of .10 or lower represented adequate fit of the model (MacCallum et al., 1996). Fit of different models was compared using chi square difference tests as well as change in CFI and RMSEA, with relatively lower RMSEA’s and higher CFI’s indicating better fit (Kline, 2005). We examined model fit indices for linear, latent, and quadratic growth curve models and determined that the latent basis growth curve model was the most suitable (Table S3).

**Appendix S5.** Correcting for multiple comparisons.

We had a priori hypotheses regarding (1) associations between the intercept and slope of earlier perceived discrimination and depression symptoms, (2) that exclusion-related brain function mediates associations between earlier perceived discrimination and depression symptoms in young adulthood, and (3) that cultural factors (ethnic pride and familism) would moderate associations between earlier perceived discrimination and exclusion-related brain function, as well as moderate associations between exclusion-related brain function and depression symptoms. We used an alpha level of .05 for all hypothesis tests. We adjusted for multiple comparisons using Bonferroni correction and considered each outcome variable as a separate hypothesis test, except when multiple operationalizations of the same construct were used. Because we had three separate hypothesis tests for depression as an outcome variable, we used a corrected alpha level of .0167. We also ran analyses to assess depression outcomes with two different subscales (anhedonic depression and general distress)—for these tests we used a corrected alpha level of .025.

**Appendix S6.** Results examining anhedonic depression and general distress.

Contrary to expectations, the intercept and slope of perceived discrimination were not significantly associated with anhedonic depression or general distress (all *p’s* > .05, Figure S3). The intercept and slope of perceived discrimination were not significantly associated with bilateral sgACC activity, and bilateral sgACC activity was not significantly associated with anhedonic depression or general distress (all *p’s* > .05, Figure S4). Given our hypothesized mediation effect, we would expect to find that perceived discrimination is associated with neural activity and neural activity is associated with anhedonic depression and general distress. However, we did not find support for either pathway. In partial support of our hypotheses, ethnic pride had a significant main effect on anhedonic depression. Specifically, higher ethnic pride in later adolescence was associated with lower anhedonic depression (β = -.21, SE = .07, *p* = .003; Figure S6). However, we did not find evidence for any of the hypothesized moderating effects of cultural factors. Specifically, earlier ethnic pride did not moderate associations between earlier perceived discrimination and bilateral sgACC brain activity, nor did later ethnic pride moderate associations between bilateral sgACC brain activity and anhedonic depression or general distress (all *p*’s > .05, Figure S6). Similarly, earlier familism did not moderate associations between earlier perceived discrimination and bilateral sgACC brain activity, nor did later familism moderate associations between bilateral sgACC brain activity and anhedonic depression or general distress (all *p*’s > .05, Figure S6).

**Appendix S7.** Results examining sensitivity analyses including only participants with usable fMRI data at both time points.

Contrary to expectations, we did not find any significant effects for sensitivity analyses examining associations between (1) the intercept and slope of earlier perceived discrimination and young adult depression symptoms (all *p’s* > .05, Figure S7), (2) the mediating effect of bilateral sgACC activity on associations between perceived discrimination in earlier adolescence and depression in young adulthood (all *p’s* > .05, Figure S7), or (3) the moderating effect of ethnic pride and familism on associations between earlier perceived discrimination, bilateral sgACC activity, and depression in young adulthood (all *p’s* > .05, Figure S7).

**Table S1.** Participant retention/attrition across the waves for the California Families Project

| Wave | Sample Size | Percent Retained |
| --- | --- | --- |
| 1 | 674 | 100% |
| 2 | 569 | 84% |
| 3 | 578 | 86% |
| 4 | 591 | 88% |
| 5 | 605 | 90% |
| 6 | 590 | 88% |
| 7 | 600 | 89% |
| 8 | 600 | 89% |
| 9 | 587 | 87% |
| 10 | 589 | 87% |
| 11 | 542 | 80% |
| 12 | 548 | 81% |
| 13 | 507 | 75% |
| 14 | 508 | 75% |

**Table S2.** Results for attrition analyses for study variables of interest.

| Variable | *t*(df) | *p* |
| --- | --- | --- |
| Time 1 Bilateral sgACC | 0.13(185) | 0.90 |
| Time 2 Bilateral sgACC | 0.50(127) | 0.62 |
| Experiences of Discrimination (Age 10) | 0.72(189) | 0.47 |
| Experiences of Discrimination (Age 11) | -0.42(177) | 0.67 |
| Experiences of Discrimination (Age 12) | -0.22(183) | 0.83 |
| Experiences of Discrimination (Age 13) | 1.74(186) | 0.08 |
| Experiences of Discrimination (Age 14) | -0.44(190) | 0.66 |
| Depression Symptoms (Age 14) | -0.52(191) | 0.60 |
| Depression Symptoms (Ages 21 & 23) | -1.99(169) | 0.05 |
| Early Ethnic Pride (Ages 10, 12, 14) | -1.09(191) | 0.28 |
| Later Ethnic Pride (Age 19) | 1.36(180) | 0.17 |
| Early Familism (Ages 10, 12, 14) | 0.13(191) | 0.90 |
| Later Familism (Age 19) | 0.75(181) | 0.45 |

**Table S3.** Growth curve model fit statistics.

| Growth Curve Model | Factor Loadings | CFI | TLI | RMSEA | χ^2^(df) | *p* |
| --- | --- | --- | --- | --- | --- | --- |
| Linear | 0, 1, 2, 3, 4 | 0.27 | 0.56 | 0.14 | 45.44 (10) | 0.00 |
| Latent Basis | 0, 0.34, 1.13, 1.11, 1 | 1.00 | 1.12 | 0.00 | 32.73 (10) | 0.00 |
| Quadratic | 0, 1, 4, 9, 16 | 0.16 | 0.16 | 0.14 | 52.54 (10) | 0.00 |

Note. CFI = comparative fit index; TLI = Tucker Lewis Index; RMSEA = root-mean-square-error of approximation.

**Table S4.** Intercept and slope variance for the discrimination variable.

| Discrimination | *σ^2^* | *p* |
| --- | --- | --- |
| Intercept | 0.05 | 0.06 |
| Slope | 0.05 | 0.08 |

**Figure S1.** Flowchart of quality control for participants.


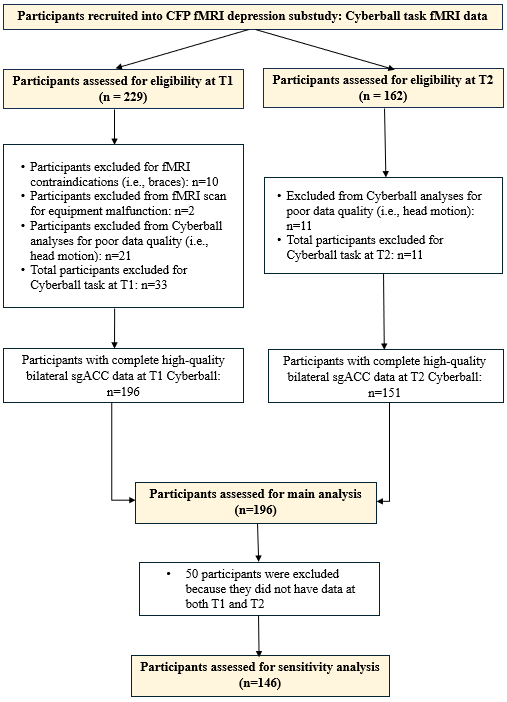


**Figure S2.** Flowchart outlining statistical analyses.

**Main Analyses (n = 196)**

Participants who had brain data available for at least one of the two time points

**Sensitivity Analyses (n = 146)**

Participants who had brain data available at both time points

Cultural factors would moderate associations between perceived discrimination and exclusion-related brain function, as well as moderate associations between exclusion-related brain function and depression

**Hypothesis 3**

Analyses were then run separately for anhedonic depression and general distress as outcomes

**Post Hoc Analyses**

**Hypothesis 1**

**Hypothesis 2**

**Latent Basis Growth Curve Model**

Examined the association between earlier experiences of perceived discrimination and exclusion-related brain function, and the association between exclusion-related brain function and depression

Examined how earlier experiences of perceived discrimination were associated with depression in young adulthood

**Latent Basis Growth Curve Model**

**Latent Basis Growth Curve Model**

Created a variable for earlier levels of ethnic pride/familism and included this variable simultaneously as a moderator of the paths from the intercept and slope of perceived discrimination to bilateral sgACC activity, and then separately tested age 19 levels of ethnic pride/familism as a moderator on the path from bilateral sgACC activity and depression

Perceived discrimination across multiple years of adolescence would be associated with depression in young adulthood

Exclusion-related brain activity would mediate associations between perceived discrimination and depression

**Figure S3.** Results of growth curve model for main analyses examining associations between earlier perceived discrimination and (A) depression symptoms as well as depression symptoms assessed by subscales for (B) anhedonic depression and (C) general distress in participants who had usable brain data available for at least one of the two time points.


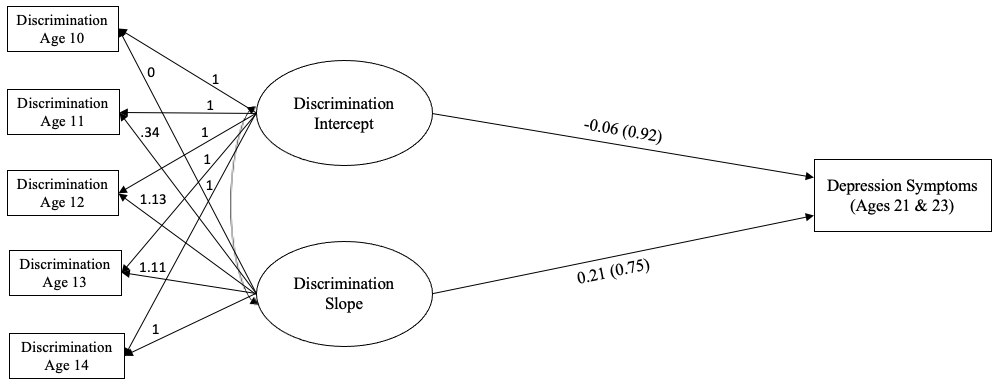
(A)


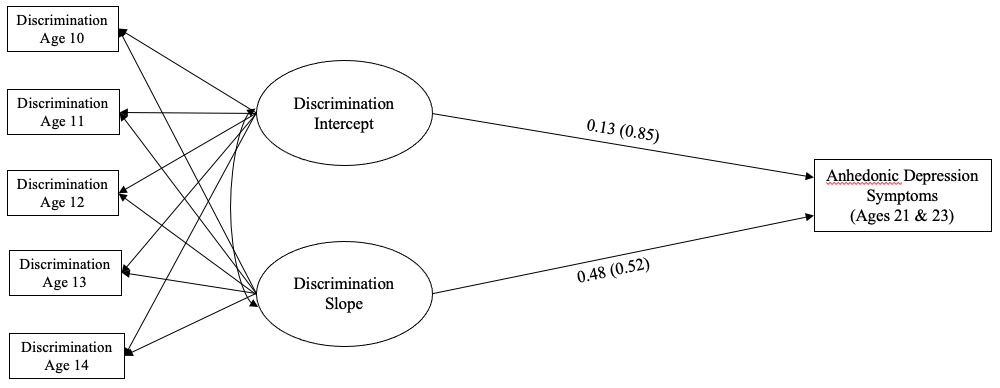
 (B)

(C)

**
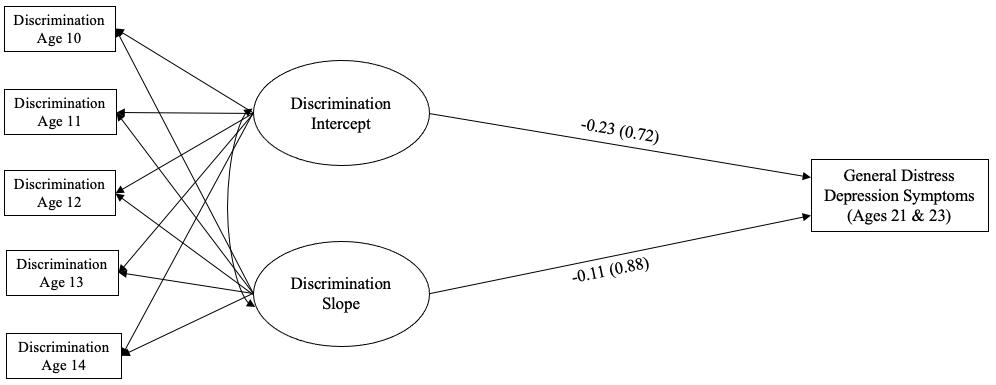
**

**Figure S4.** Results of growth curve model for analyses examining associations between earlier perceived discrimination, averaged brain activity in later adolescence, and depression symptoms in young adulthood. (A) Association between earlier perceived discrimination, later adolescent bilateral sgACC brain activity, and young adult depression symptoms. (B) Association between earlier perceived discrimination, later adolescent bilateral sgACC brain activity, and young adult anhedonic depression. (C) Association between earlier perceived discrimination, later adolescent bilateral sgACC brain activity, and young adult general distress. These analyses were tested in participants who had usable brain data available for at least one of the two time points.


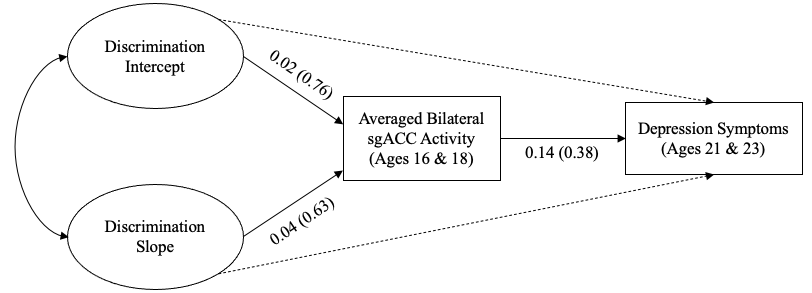
(A)

(B)


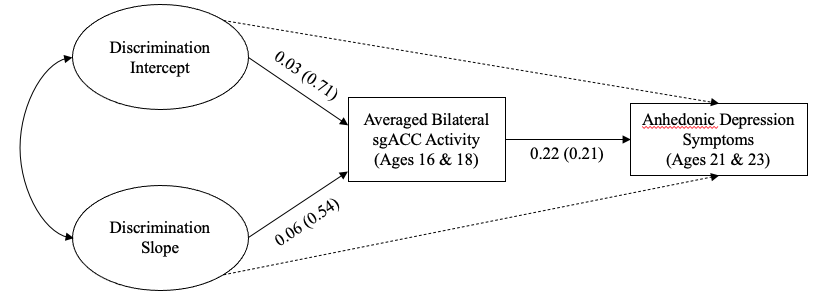


**
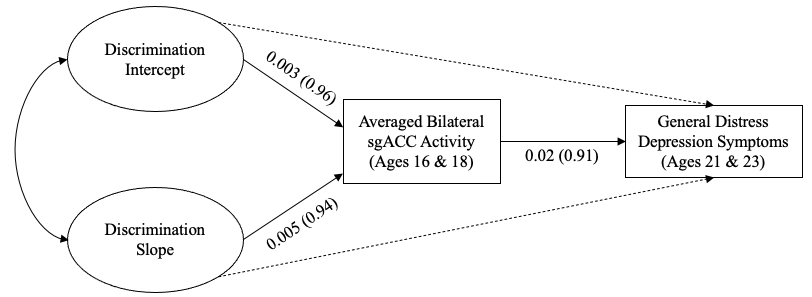
** (C)

**Figure S5**. Results of growth curve model for exploratory analyses examining associations between changes in bilateral sgACC brain activity and depression symptoms in participants who had usable brain data at both time points.


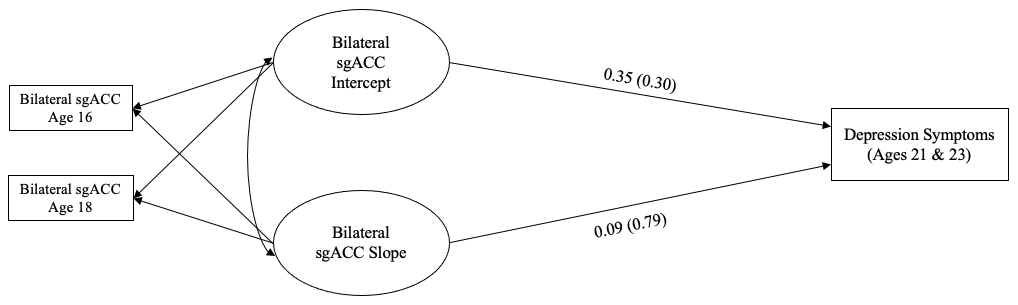


**Figure S6.** Results of growth curve model for analyses examining associations between earlier perceived discrimination, averaged bilateral sgACC brain activity in later adolescence, and depression in young adulthood with (A) familism as a moderating variable as well as, analyses examining associations between earlier perceived discrimination, averaged bilateral sgACC brain activity in later adolescence, and anhedonic depression in young adulthood with (B) ethnic pride and (C) familism as moderating variables and, analyses examining associations between earlier perceived discrimination, averaged bilateral sgACC brain activity in later adolescence, and general distress in young adulthood, with (D) ethnic pride and (E) familism as moderating variables. These analyses were run in participants who had usable brain data available for at least one of the two time points.


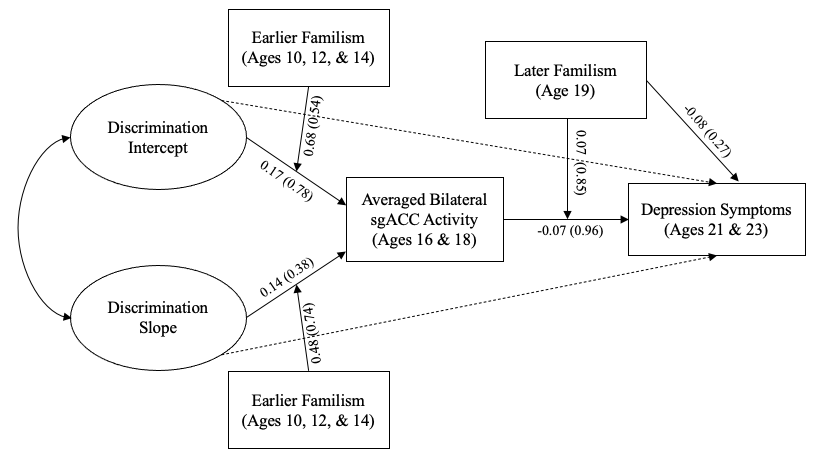
(A)


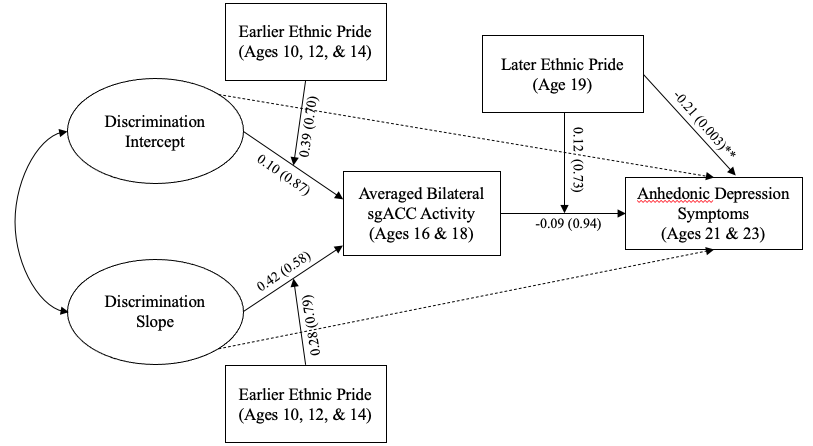
(B)


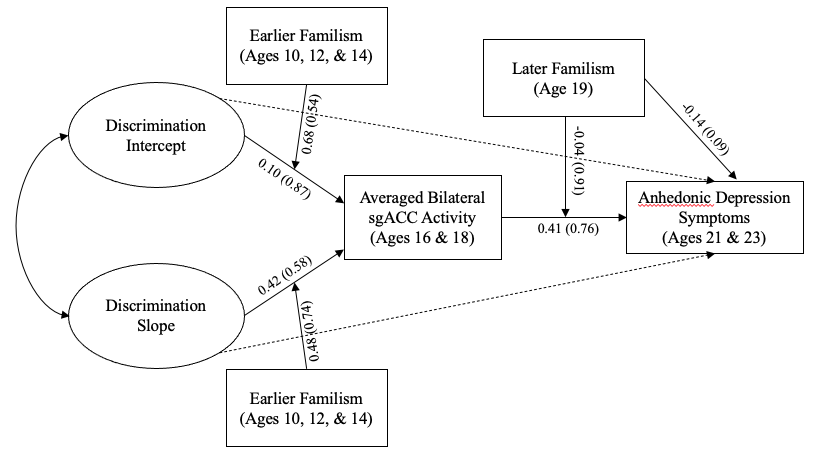
(C)


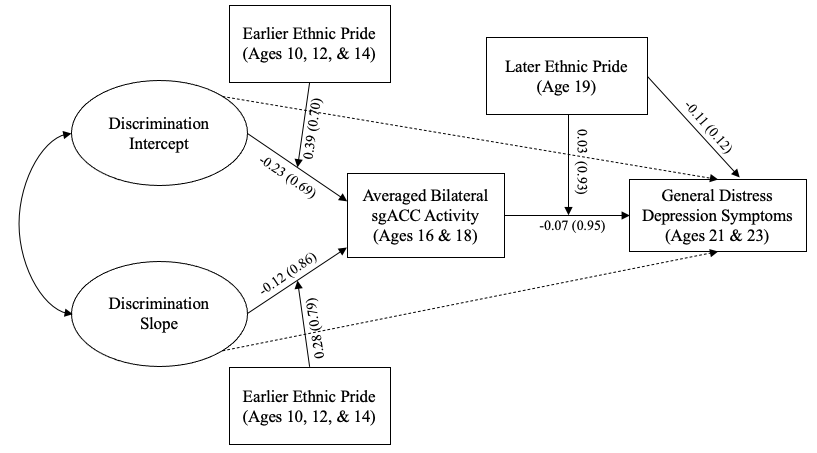
(D)


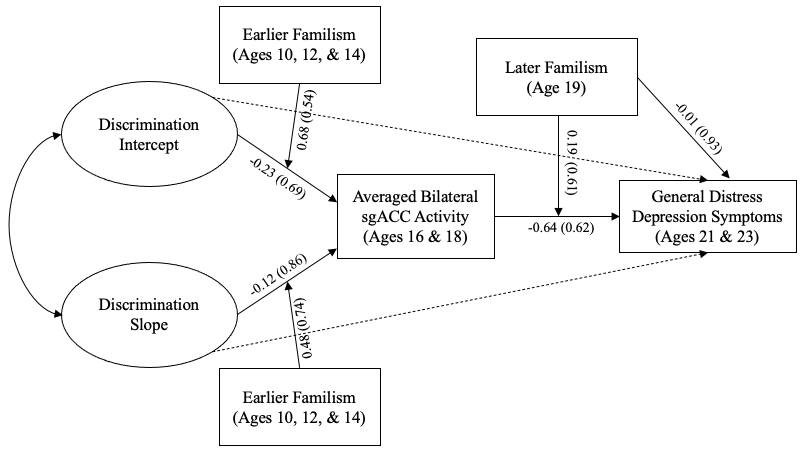
(E)

**Figure S7**. Results of growth curve model for sensitivity analyses examining (A) the association between earlier perceived discrimination and depression symptoms, (B) the association between earlier perceived discrimination, later adolescent bilateral sgACC brain activity, and young adult depression symptoms, and associations between earlier perceived discrimination, averaged brain activity in later adolescence, and depression symptoms in young adulthood, with (C) ethnic pride and (D) familism as moderating variables in participants who had usable brain data at both time points.


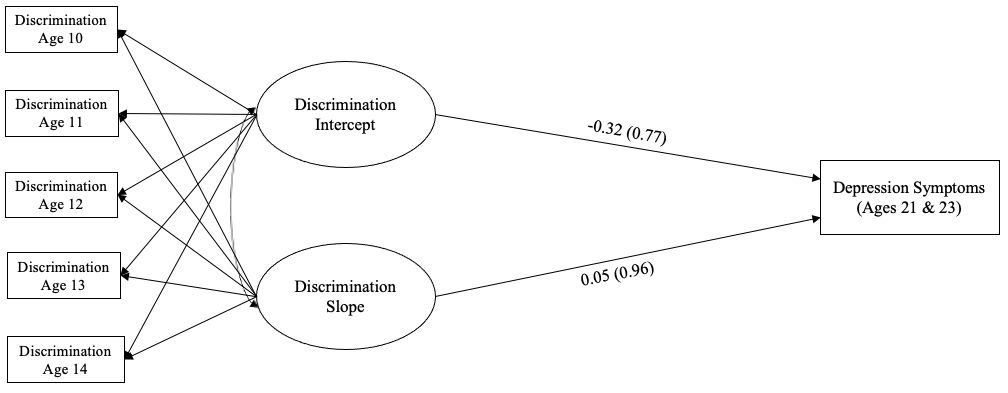
(A)

(B)


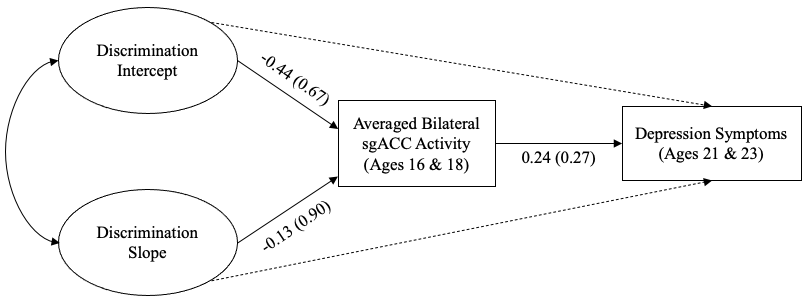


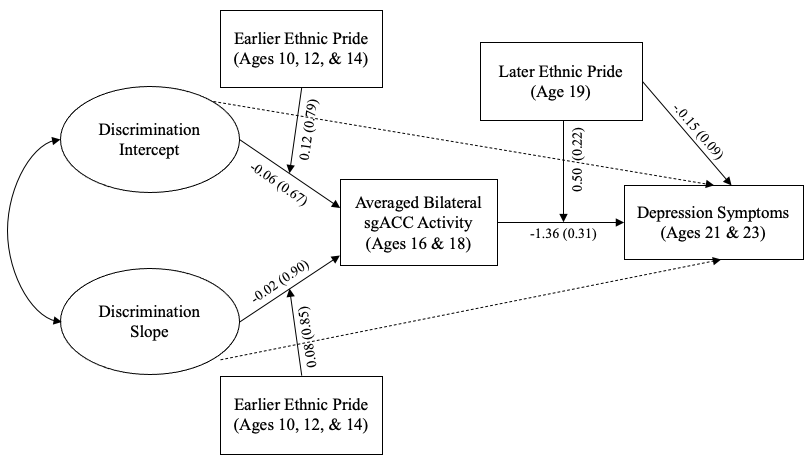
(C)


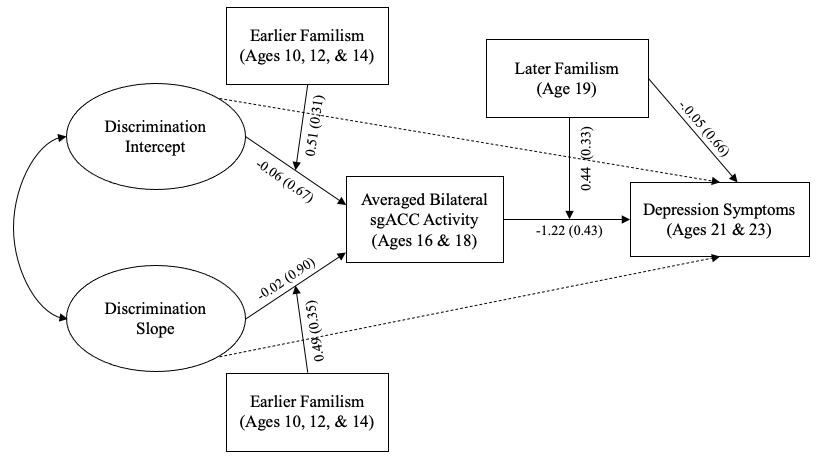
(D)

**Supplementary References**

Bentler, P. M. (1990). Comparative fit indexes in structural models. *Psychological*

*Bulletin*, *107*(2), 238–246. https://doi.org/10.1037/0033-2909.107.2.238

Bollen, K. A. (1989). *Structural equations with linear variables.* John Wiley &

Sons. https://doi.org/10.1002/9781118619179

Kline, R. (2005). Principles and practice of Structural Equation Modeling (2nd ed.). New York:

Guilford.

MacCallum, R. C., Browne, M. W., & Sugawara, H. M. (1996). Power analysis and

determination of sample size for covariance structure modeling. *Psychological Methods, 1*(2), 130–149. https://doi.org/10.1037/1082-989X.1.2.130

Meyer, M. L., Williams, K. D., & Eisenberger, N. I. (2015). Why social pain can live on:

Different neural mechanisms are associated with reliving social and physical pain. PloS one, 10(6), e0128294. https://doi.org/10.1371/journal.pone.0128294

Schriber, R. A., Rogers, C. R., Ferrer, E., Conger, R. D., Robins, R. W., Hastings, P. D., &

Guyer, A. E. (2018). Do hostile school environments promote social deviance by shaping neural responses to social exclusion?*Journal of Research on Adolescence, 28*(1), 103-120. https://doi.org/10.1111/jora.12340

Shaffer, D., Fisher, P., Lucas, C. P., Dulcan, M. K., & Schwab-Stone, M. (2000). NIMH

Diagnostic Interview Schedule for Children Version IV (NIMH DISC-IV): Description, differences from previous versions, and reliability of some common diagnoses. Journal of the American Academy of Child & Adolescent Psychiatry, 39(1), 28-38. https://doi.org/10.1097/00004583-200001000-00014

Steiger, J. H. (1990). Structural model evaluation and modification: An interval estimation

approach. *Multivariate Behavioral Research*, *25*(2), 173–180. https://doi.org/10.1207/s15327906mbr2502_4

Vogt, B. A., Berger, G. R., & Derbyshire, S. W. G. (2003). Structural and functional dichotomy

of human mid cingulate cortex. The European Journal of Neuroscience, 18, 3134–3144. https://doi.org/10.1111/j.1460-9568.2003.03034.x
